# Alternative ion channel splicing in mesial temporal lobe epilepsy and Alzheimer's disease

**DOI:** 10.1186/gb-2007-8-3-r32

**Published:** 2007-03-07

**Authors:** Erin L Heinzen, Woohyun Yoon, Michael E Weale, Arjune Sen, Nicholas W Wood, James R Burke, Kathleen A Welsh-Bohmer, Christine M Hulette, Sanjay M Sisodiya, David B Goldstein

**Affiliations:** 1 Institute for Genome Sciences and Policy, Center for Population Genomics and Pharmacogenetics, Duke University, Durham, NC 27710, USA; 2 Department of Clinical and Experimental Epilepsy, Institute of Neurology, Queen Square, London WC1N 3BG, UK; 3 Department of Molecular Neuroscience, Institute of Neurology, Queen Square, London WC1N 3BG, UK; 4 Joseph and Kathleen Bryan Alzheimer's Disease Research Center, Duke University, Durham, NC 27710, USA

## Abstract

**Background:**

Alternative gene transcript splicing permits a single gene to produce multiple proteins with varied functions. Bioinformatic investigations have identified numerous splice variants, but whether these transcripts are translated to functional proteins and the physiological significance of these alternative proteins are largely unknown. Through direct identification of splice variants associated with disease states, we can begin to address these questions and to elucidate their roles in disease predisposition and pathophysiology. This work specifically sought to identify disease-associated alternative splicing patterns in ion channel genes by comprehensively screening affected brain tissue collected from patients with mesial temporal lobe epilepsy and Alzheimer's disease. New technology permitting the screening of alternative splice variants in microarray format was employed. Real time quantitative PCR was used to verify observed splice variant patterns.

**Results:**

This work shows for the first time that two common neurological conditions are associated with extensive changes in gene splicing, with 25% and 12% of the genes considered having significant changes in splicing patterns associated with mesial temporal lobe epilepsy and Alzheimer's disease, respectively. Furthermore, these changes were found to exhibit unique and consistent patterns within the disease groups.

**Conclusion:**

This work has identified a set of disease-associated, alternatively spliced gene products that represent high priorities for detailed functional investigations into how these changes impact the pathophysiology of mesial temporal lobe epilepsy and Alzheimer's disease.

## Abstract 2

A novel microarray technology that permits the screening of alternative splice variants identifies disease-associated alternative splicing patterns in ion channel genes of patients with mesial temporal lobe epilepsy and Alzheimers disease.

## Background

The complexity of the genome lies not only in the many genes comprising it, but also in the many levels of processing that influence the proteins that are produced and their abundance. One key site of regulation is the splicing of precursor RNAs to their associated mRNA transcripts. This process alone allows a single gene to have multiple different mRNA transcripts, producing proteins that may differ substantially from one another, even to the extent of having opposing effects [1]. Overall, however, little is known about the functional differences amongst the alternative proteins produced from the same gene. Because the functional characterization of proteins can be laborious, it would be useful to be able to prioritize alternative transcripts more likely to have biological significance. One direction for prioritization is on the basis of association with human disease.

Alternative splicing of key genes generates alternative proteins that contribute to several prominent human diseases, for example, the spinal motor neuron protein in spinal muscular atrophy [2], cardiac troponin T, insulin receptor, myotubularin-related 1, and other proteins in myotonic dystrophies [3-5], and the tau protein in frontotemporal dementia and Alzheimer's disease [3,4] (other examples are reviewed extensively in [5]). Furthermore, alternative splicing of a sodium channel gene, *SCN1A*, has also recently been associated with altered response to antiepileptic medications [6]. There are potentially many more undetected examples of splicing alterations associated with disease pathophysiology and drug response variation in humans.

Studies of alternative splicing have usually been restricted to a single gene or small gene family. To date, there are only a few reports of splice variation screens in human disease and none has been reported for any central nervous system disease. Recently, new technology has become available that allows for the comprehensive investigation of alternative splicing through the use of splice variant microarrays. This technology uses probes in a microarray format and screens for unique exon-exon junctions specific to a particular splicing event [7-10]. Here we applied this systematic approach to assess the relationship between alternative splicing and two common and important neurological conditions, with the aim of identifying alternative splicing patterns of potential relevance to human disease.

Mesial temporal lobe epilepsy (mTLE) and Alzheimer's disease (AD) are highly complex neurological diseases characterized by aberrant neuronal excitation and neurodegeneration. While the pathological processes differ substantially, both diseases exhibit pathophysiology linked to ion channel activity. Seizure activity characteristic of epilepsy is the result of a dysregulation of inhibitory and excitatory neuronal signaling largely controlled by ion channel activity [11]. Likewise, abnormal ion channel function also has been associated extensively with AD. AD-related neurodegeneration is believed to be, in part, caused by the overactivation of N-methyl-D-aspartate receptor activation and subsequent increases in intracellular calcium, oxidative stress, and neurodegeneration [12]. Other ion channels, including glutamate receptors, nicotinic cholinergic receptors, and calcium and potassium channels, also have been implicated in AD pathophysiology [13-18]. Little information exists regarding the impact of splicing variation of ion channel genes on mTLE and AD. Our work sought to comprehensively evaluate ion channel splice variation in these two neurological diseases using a microarray format (ExonHit Therapeutics). We evaluated 1,665 known and potential splice events across 287 ion channel genes in human brain tissue samples collected from patients with AD and mTLE. In addition to identifying disease-associated splicing variation, a secondary aim of this work was to assess the reliability of the array-based identification of splicing changes through the use of real time PCR (rtPCR) to validate associations detected using the high-throughput platform.

## Results

### Mesial temporal lobe epilepsy

Following our initial screen of 1,665 possible alternative splicing events, a total of 221 splicing changes were identified as statistically significantly changed in mTLE samples, with *p *< 0.05, when comparing splice variant ratio (SVR) values calculated using equation 1 (see Materials and methods). Selected statistically significant events representing a range of *p *values were chosen for rtPCR confirmation. Of 13 splice array-identified alternative splicing events with an associated *p *value of less than 0.05, 9 were verified using rtPCR in a larger sample size. Evaluation of discrete groups of *p *value ranges revealed increased success rates with lower *p *values (0.02 <* p *< 0.05, 0% success (two events evaluated); 0.01 <* p *< 0.02, 70% success (seven events evaluated); *p *< 0.01, 100% success (3 events evaluated)). In order to report changes most likely to be real, only events that had a *p *value in splice array analyses of less than 0.02 are reported (Table 1). With this additional filter, a total of 126 alternative splicing events were observed in brain tissue collected from mTLE patients. As additional confirmation, four events not identified as changed in the splice array screen (*p *> 0.05) were confirmed not to be changed using rtPCR. The compiled list of rtPCR confirmed mTLE-associated alternative splicing events are included in Table 2.

**Table 1:** List of ion channel genes exhibiting alternative splicing in patients with mTLE

Genes by gene class	GenBank accession number (reference transcript*)	GenBank accession number (variant transcript*)	Alternative splicing event: change of mRNA transcript composition in epilepsy^†^	Alternatively spliced region	Splice array *p *value
**Calcium channels**					
*CACNA1A*	NM_023035	U79668	ES: 26% ↓ variant/reference ratio	EXON 48	0.002
	NM_023035	CQ723237	ASA: 24% ↓ reference/total ratio	EXON 23	0.003
	NM_023035	BE972738	NE: 36% ↓ variant/total ratio	3' downstream	<0.001
*CACNA1C*	NM_000719	CQ722696	ASD: 94% ↓ variant/reference ratio	EXON 2	<0.001
	NM_000719	CQ722691	ASD: 23% ↓ variant/reference ratio	EXON 24	0.017
*CACNA1H*	NM_021098	CQ730788	ASA: 25% ↓ variant/reference ratio	EXON 34	<0.001
*CACNA1I*	NM_001003406	AX068892	NE: 36% ↓ variant/reference ratio	INTRON 1	0.012
	NM_001003406	AX068892	NE: 23% ↓ variant/reference ratio	INTRONS 20/21	0.009
*CACNA2D1*	NM_000722	BG211441	ES: 28% ↑ reference/total ratio	EXON 8	0.003
*CACNB1*	NM_000723	NM_199247	NE: 28% ↑ variant/reference ratio	INTRON 6	<0.001
	NM_000723	BP316738	NE: 28% ↑ variant/reference ratio	INTRON 6	0.002
*CACNB2*	NM_201596	AF465485	ES: 20% ↓ variant/reference ratio	EXON 7	0.020
*CACNB3*	NM_000725	AK122911	NEs: 28% ↓ variant/total ratio	5' upstream	<0.001
*CACNB4*	NM_000726	AY054985	NE: 31% ↑ variant/reference ratio	INTRON 2	0.012
*CACNG4*	NM_014405	AW134993	PIED: 45% ↑ reference/total ratio	EXON 4	0.009
	NM_014405	AI675178	PIED: 41% ↓ variant/reference ratio	EXON 4	0.003
					
**Chloride channels**					
*CLCN2*	NM_004366	BC021578	ES: 38% ↓ reference/total ratio	EXON 13	<0.001
*CLCN3*	NM_001829	CQ732096	ASA: 22% ↓ variant/reference ratio	EXON 2	0.004
	NM_001829	CQ736554	NE: 32% ↑ reference/total ratio	INTRON 12	0.012
*CLCN6*	NM_001286	BC050457	ASA: 16% ↓ variant/reference ratio	EXON 20	0.016
*CLCN7*	NM_001287	AK096963	IR: 36% ↓ variant/reference ratio	INTRON 18	<0.001
	NM_001287	BQ920088	ASA: 21% ↓ variant/reference ratio	EXON 12	0.004
*CLIC5*	NM_016929	BC039380	ASA: 20% ↓ reference/total ratio	EXON 5	0.013
					
**Sodium channels**					
*SCN2A2*	NM_021007	BC029489	ES: 23% ↑ reference/total ratio	EXON 1	0.006
*SCN9A*	NM_002977	BG108767	ASD: 26% ↓ variant/reference ratio	EXON 16	0.003
*SCNN1A*	NM_001038	BF033087	PIED: 21% ↓ variant/reference ratio	EXON 13	<0.001
*SCNN1D*	NM_002978	AX230571	NE: 52% ↑ variant/total ratio	INTRON 2	0.012
	NM_002978	AK127357	NE: 77% ↑ variant/total ratio	5' upstream	0.002
					
**Potassium channels**					
*KCNAB2*	NM_003636	BG720519	ES: 35% ↑ variant/reference ratio	EXON 1	0.014
	NM_003636	BM823724	NEs: 19% ↓ variant/total ratio	5' upstream	0.015
	NM_003636	CA495339	IR: 24% ↓ reference/total ratio	INTRON 11	0.020
*KCNC3*	NM_004977	AK127492	ASD: 27% ↓ variant/reference ratio	EXON 5	0.020
*KCNH5*	NM_172376	NM_139318	NE: 24% ↑ variant/total ratio	3' downstream	<0.001
*KCNJ1*	NM_153767	NM_000220	NE: 27% ↓ variant/reference ratio	INTRON 3	<0.001
*KCNJ6*	NM_002240	CQ738104	NEs: 24% ↓ variant/total ratio	INTRON 2	0.017
*KCNJ15*	NM_170736	NM_002243	NE: 108% ↑ variant/total ratio	5' upstream	<0.001
	NM_170736	NM_170737	NE: 2.5x ↑ reference/total ratio	INTRON 2	<0.001
	NM_170736	CQ732921	NEs: 42% ↓ variant/reference ratio	INTRON 2	0.005
	NM_170736	BM544058	NE: 98% ↑ variant/total ratio	5' upstream	<0.001
	NM_170736	BF105170	NE: 78% ↑ variant/total ratio	5' upstream	0.003
	NM_170736	BI518753	NE: 2.5x ↑ reference/total ratio	INTRON 2	<0.001
*KCNK1*	NM_002245	AV733795	PIED: 34% ↓ variant/reference ratio	EXON 2	0.010
	NM_002245	BG699040	NE: 45% ↓ variant/total ratio	INTRON 1	0.009
	NM_002245	BF212472	NEs: 23% ↓ variant/total ratio	INTRON 1	0.017
	NM_002245	BU661246	NEs: 48% ↓ variant/total ratio	INTRON 1	<0.001
*KCNK2*	NM_014217	BU956092	ES: 62% ↑ reference/total ratio	EXON 4	0.008
*KCNK4*	NM_016611	NM_033310	NE: 102% ↑ variant/total ratio	5' upstream	0.005
*KCNK12*	NM_022055	AX302031	NEs: 20% ↓ variant/total ratio	INTRON 1	0.014
*KCNMA1*	NM_002247	CQ870200	NE: 24% ↓ variant/reference ratio	EXON 20	0.006
	NM_002247	CQ870204	PIED: 32% ↓ variant/reference ratio	3' downstream	0.020
	NM_002247	BG185868	NE: 28% ↓ variant/reference ratio	EXON 9	0.014
*KCNN2*	NM_021614	NM_170775	NE: 26% ↓ variant/total ratio	INTRON 3	0.002
*KCNQ2*	NM_004518	AY358189	NE: 28% ↓ variant/reference ratio	INTRON 7	0.018
	NM_004518	BG772772	NEs: 23% ↓ variant/reference ratio	INTRON 7	0.018
*HCN3*	NM_020897	CQ715247	EsS: 15% ↓ variant/reference ratio	EXONS 3-4	0.012
*SLICK*	NM_198503	CQ728754	EsS: 28% ↓ reference/total ratio	EXONS 22-25	0.019
	NM_198503	CQ728754	ES: 57% ↑ variant/reference ratio	EXON 15	0.003
					
**GABA receptors**					
*GABRA2*	NM_000807	CD014113	ES: 26% ↑ reference/total ratio	EXON 8	<0.001
	NM_000807	CD014116	EsS: 29% ↑ reference/total ratio	EXONS 4-9	<0.001
	NM_000807	CD014112	EsS: 35% ↑ reference/total ratio	EXON 4	<0.001
	NM_000807	CD014107	EsS: 31% ↑ reference/total ratio	EXONS 7/8	<0.001
	NM_000807	CD014110	EsS: 36% ↑ reference/total ratio	EXONS 6-8	<0.001
	NM_000807	CD014109	EsS: 40% ↑ reference/total ratio	EXONS 6/7	<0.001
*GABRA3*	NM_000808	AX897950	ES: 17% ↓ reference/total ratio	EXON 7	0.007
*GABRG2*	NM_198904	NM_198903	ASA: 22% ↑ reference/total ratio	EXON 4	0.005
*GABRR1*	NM_002042	CB959800	NEs: 17% ↓ variant/total ratio	5' upstream	0.007
					
**Ionotropic glutamate receptors**					
*GRIA1*	NM_000827	A46050	NE: 34% ↓ variant/reference ratio	INTRON 14	0.012
	NM_000827	M64752	NE: 39% ↓ variant/reference ratio	INTRON 14	0.008
*GRIA2*	NM_000826	BC010574	NE: 44% ↓ variant/reference ratio	INTRON 13	0.007
	NM_000826	AX147452	NE: 61% ↓ variant/reference ratio	INTRON 13/EXON14	0.005
	NM_000826	AV748963	NE: 44% ↓ variant/reference ratio	EXON 14	0.015
*GRIA3*	NM_007325	NM_181894	NEs: 19% ↑ variant/reference ratio	INTRON 2	0.006
*GRIK2*	NM_021956	CQ715784	ASD: 65% ↓ reference/total ratio	EXON 10	<0.001
*GRIK4*	NM_014619	CQ869986	NEs: 29% ↓ variant/total ratio	5' upstream	0.006
	NM_014619	CQ715345	NE: 31% ↓ variant/total ratio	INTRON 4	0.015
	NM_014619	CQ715345	ES: 23% ↑ reference/total	EXON 7	0.005
*GRIK5*	NM_002088	CQ719647	ASD: 24% ↓ reference/total ratio	EXON 16	0.008
	NM_002088	AX665460	ASD: 23% ↓ variant/reference ratio	EXON 18	0.004
	NM_002088	CN420154	EsS: 27% ↓ variant/reference	EXONS 12-14	0.020
					
**FXYD domain ion transport regulators**					
*FXYD1*	NM_021902	BQ181273	IR: 51% ↓ variant/reference ratio	INTRON 7	<0.001
*FXYD6*	NM_022003	AX430335	NE: 31% ↓ variant/total ratio	5' upstream	<0.001
	NM_022003	AX892598	NE: 35% ↓ variant/total ratio	5' upstream	<0.001
	NM_022003	BC018652	NEs: 38% ↓ variant/reference ratio	EXON 1/INTRON 1	<0.001
	NM_022003	AL832811	NE: 33% ↓ variant/reference ratio	INTRON 1	<0.001
	NM_022003	AW270073	ES: 34% ↓ variant/reference ratio	EXON 9	<0.001
	NM_022003	BI823239	NE: 48% ↓ variant/reference ratio	INTRON 1	<0.001
	NM_022003	BF527041	NE: 36% ↓ variant/reference ratio	INTRON 1	0.002
	NM_022003	BP372334	NE: 45% ↓ variant/reference ratio	INTRON 1	<0.001
	NM_022003	BE263758	NE: 37% ↓ variant/reference ratio	EXON 2	<0.001
	NM_022003	BX488702	ASA: 50% ↓ variant/reference ratio	INTRON 2	<0.001
	NM_022003	BI598749	NE: 40% ↓ variant/reference ratio	INTRON 1	0.006
*FXYD7*	NM_022006	CQ722304	ES: 21% ↓ variant/reference ratio	EXON 4	0.007
					
**Transient receptor potential cation channels**					
*TRPA1*	NM_007332	BF570694	ASD: 37% ↓ reference/total ratio	EXON 25	<0.001
*TRPC4*	NM_016179	AF421362	ES: 24% ↓ reference/total ratio	EXON 3	0.004
*TRPM1*	NM_002420	AX480882	ASD:43% ↓ variant/reference ratio	EXON 17	<0.001
*TRPM7*	NM_017672	CQ728707	EsS: 35% ↑ variant/reference ratio	EXONS 7-11	<0.001
*TRPV1*	NM_080704	NM_080705	NE: 31% ↑ variant/total ratio	INTRON 1	0.007
	NM_080704	AX686983	ES: 35% ↑ variant/reference ratio	EXON 8	0.005
*TRPV2*	NM_016113	BQ645005	ASD: 25% ↓ variant/reference ratio	EXON 15	0.005
					
**Glycine receptors**					
*GLRB*	NM_000824	CD013911	ES: 31% ↑ variant/reference ratio	EXON 9	0.004
					
**Inositol triphosphate receptors**					
*ITPR1*	NM_002223	CQ727353	EsS: 22% ↑ variant/reference ratio	EXONS 52-54	0.020
					
**Purinergic receptors**					
*P2RX1*	NM_002558	BM469621	NE: 57% ↑ variant/total ratio	INTRON 3	<0.001
	NM_002558	BP372168	ASD: 17% ↑ reference/total ratio	EXON 1	0.002
*P2RXL1*	NM_005446	BC064805	ASD: 24% ↑ variant/reference ratio	EXON 1	0.013
					
**Cyclic nucleotide gated channels**					
*CNGA1*	NM_000087	CN366905	NEs: 20% ↓ variant/total ratio	5' upstream	0.011
*CNGB3*	NM_019098	AI150392	ES: 19% ↓ variant/total ratio	EXON 18	0.016
					
**Amiloride-sensitive cation channel**					
*ACCN2*	NM_020039	BC028722	NE: 19% ↓ variant/reference	INTRON 3	0.017
	NM_020039	AX683970	NE: 23% ↓ variant/reference	INTRON 3	0.019
					
**Two pore segment channels**					
*TPCN1*	NM_017901	CQ729206	ASA: 27% ↑ variant/reference ratio	EXON 10	<0.001
*TPCN2*	NM_139075	AW178475	NE: 87% ↑ variant/total ratio	INTRON 9	<0.001
					
**Mucolipin**					
*MCOLN1*	NM_020533	BQ723075	ASD: 17% ↓ variant/reference ratio	EXON 2	<0.001
	NM_020533	CA489568	ASD: 69% ↑ variant/reference ratio	EXON 7	0.011
					
**Ryanodine receptors**					
*RYR3*	NM_001036	CQ730808	ES: 28% ↑ variant/total ratio	EXON 84	0.017
					
**Miscellaneous other ion channels/interacting proteins**					
*C6orf69*	NM_173562	BC023525	EsS: 33% ↑ reference/total ratio	EXONS 3-5	0.003
*KCNIP2 *(potassium channel interacting protein)	NM_014591	NM_173191	ASA: 37% ↑ variant/reference ratio	EXON 3	0.004
	NM_014591	NM_173193	EsS: 19% ↑ variant/reference ratio	EXONS 2-3	0.018
	NM_014591	NM_173197	ES: 9% ↓ reference/total ratio	EXON 7	0.015
*MLC1 *(megalencephalic leuko-encephalopathy with subcortical cysts)	NM_015166	BX451200	ASD: 31% ↓ variant/reference ratio	EXON 8	0.009
*PKD1L2 *(polycystic kidney disease 1-like)	NM_052892	CQ741519	ES: 82% ↑ variant/reference ratio	EXON 7	0.006
*PKD2L2 *(polycystic kidney disease 2like)	NM_014386	AF182034	NEs: 38% ↑ variant/total ratio	INTRON 13/3' downstream	0.005
*SH3KBP1 *(SH3KBP1 binding protein)	NM_138392	BX366064	ASD: 26% ↓ variant/reference ratio	EXON 10	0.002
	NM_138392	AL523485	ASD: 26% ↑ reference/total ratio	EXON 13	0.005
*VGCNL1 *(voltage-gated channel)	NM_052867	BM556576	NE: 31% ↓ variant/total ratio	INTRON 7	<0.001
	NM_052867	AK094669	EsS: 28% ↓ reference/total ratio	INTRON 12	0.019

*Reference transcript refers to traditionally spliced gene product, while the variant refers to the alternatively spliced mRNA transcript. ^†^Change was quantified using splice array samples (control versus mTLE temporal cortex, *n *= 10/group). The ratio selected to quantify an event was determined based on best available probe choice (see Materials and methods section). ASA, alternative splice acceptor site; ASD, alternative splice donor site; ES, exon skipped; EsS, exons skipped; IR, intron retention; NE, novel exon; NEs, novel exons; PIED, partial internal exon deletion.

**Table 2:** List of alternative splicing events confirmed using rtPCR

Gene	GenBank accession number (reference transcript*)	GenBank accession number (variant transcript*)	Alternative splicing event: change of mRNA transcript composition in disease state^†^	Alternatively spliced region	rtPCR *p *value
**mTLE**					
*CACNB1*	NM_000723	NM_199247	NE: 65% ↑ variant/reference ratio	INTRON 6	0.035
*CACNB4*	NM_000726	AY054985	NE: 4.2x ↑ variant/reference ratio	INTRON 2	0.027
*CLCN7*	NM_001287	AK096963	IR: 65% ↓ variant/total ratio	INTRON 18	<0.001
*FXYD6*	NM_022003	BP372334	NE: 22% ↓ variant/reference ratio	INTRON 1	<0.001
*GRIA1*	NM_000827	A46050	NE: 62% ↓ variant/reference ratio	INTRON 14	0.002
*KCNK1*	NM_002245	AV733795	PIED: 13% ↓ variant/total ratio	EXON 2	<0.001
*KCNQ2*	NM_004518	AY358189	NE: 34% ↓ variant/total ratio	INTRON 7	<0.001
*MCOLN1*	NM_020533	CA489568	ASD: 47% ↑ variant/reference ratio	EXON 7	<0.001
					
**AD**					
*CACNA1G*	NM_018896	NM_198376	PIED: 67% ↓ variant/total ratio	EXON 36	<0.001
*GABRA6*	NM_000811	AK090735	IR: 30% ↓ variant/reference ratio	INTRON 6	0.007
*GRIA1*	NM_000827	A46050	NE: 2.5x ↑ variant/reference ratio	INTRON 14	0.001
*KCNAB1*	NM_172160	NM_172159	NE: 75% ↓ variant/reference ratio	INTRON 1	0.004
*KCNN1*	NM_002248	BM718136	ASD: 25% ↓ variant/reference ratio	EXON 6	<0.001
*KCNN2*	NM_021614	BG769522	ES: 85% ↓ variant/reference ratio	EXON 3	0.002
*MCOLN1*	NM_020533	CA489568	ASD: 19% ↑ variant/reference ratio	EXON 7	0.016

*Reference transcript refers to traditionally spliced gene product, while the variant refers to the alternatively spliced mRNA transcript. ^†^Splice array determined results were confirmed using quantitative rtPCR in a larger sample size (control (*n *= 28-29) versus mTLE neocortex (*n *= 43); control (*n *= 27) versus AD (*n *= 31) temporal cortex and cerebellum). ASA, alternative splice acceptor site; ASD, alternative splice donor site; ES, exon skipped; EsS, exons skipped; IR, intron retention; NE, novel exon; NEs, novel exons; PIED, partial internal exon deletion.

Our splice array studies revealed an mTLE-associated splicing change in *CACNA1B *(*p *= 0.017, variant GenBank: M94173). This particular event was randomly selected for rtPCR confirmation, and we observed a change opposite that detected with the splice array. This likely occurred due to the presence of unknown splicing events that were being detected either by the splice array probes, or possibly by the rtPCR assay probes. Due to the uncertainty linked to this event, we deemed this splicing change in our studies as an event that rtPCR failed to confirm and, therefore, it is not included in Table 1. Given our results in our rtPCR confirmation analyses of array-detected events, we believe this occurs relatively infrequently, but this does bring to light a shortcoming of the splice array technology in accurately detecting complex series of alternative splicing events that occur in a gene region.

Nearly 25% of all genes represented on the splice variant microarray were alternatively spliced in mTLE compared to control. Following normalization of the number of genes found to be alternatively spliced to the total number of genes in the gene class, calcium channels, chloride channels, and glutamate receptors were found to exhibit the most mTLE-associated alternative splicing events, with nearly 30% of genes in these classes found to be alternatively spliced in this disease state.

**Figure 1:**
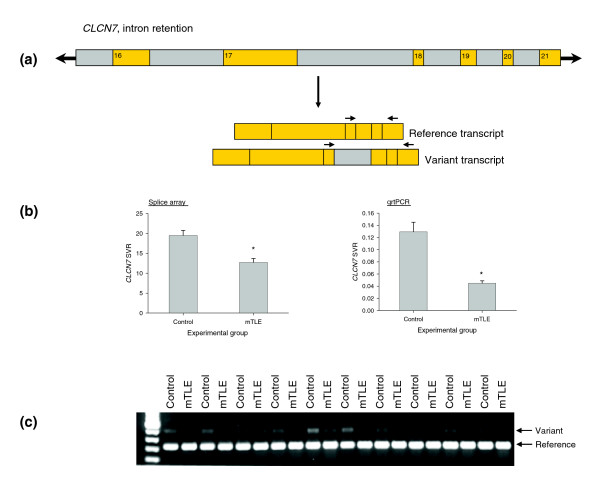
Representative mTLE-associated alternative splicing event identified using splice array technology. **(a) **Schematic of *CLCN7 *alternative splicing event associated with mTLE. Exons are shown in orange and intronic regions are shown in gray. **(b) **Data collected for the *CLCN7 *alternative splice event in control and mTLE brain tissue samples using the splice array technology (left) and quantitative rtPCR (qrtPCR, right). Data are presented as mean ± standard error of the mean; *p < 0.05 when compared to control, Student's *t*-test. **(c) **rtPCR confirmation of the pattern of transcript expression in brain tissue collected from ten subjects from each group. Both reference and variant transcript forms were amplified using the following primer sequences (indicated in the figure by arrows above mRNA transcripts): CLCN7F-GGCAAATACGCCCTGATG, CLCN7R-CTCAGCACGTCCACAATGAC.

A representative mTLE-associated alternative splicing event is shown in Figure 1. This selected event in the *CLCN7 *gene results in the inclusion of an intronic sequence located between exons 17 and 18 (Figure 1a). Both the splice array data and the rtPCR confirmation revealed a net reduction in the variant form containing the intronic sequence in mTLE brain tissue samples (Figure 1b). Furthermore, with reverse transcriptase amplification of the transcript variants in selected brain tissue samples, we show both the presence of the two transcript forms and the effects of the disease state on reducing the variant splice form of *CLCN7 *(Figure 1c).

### Alzheimer's disease

For AD, 1,479 potential splicing events passed the criteria on probe quality for the control 2 versus AD comparison. Using the SVR ratios shown in equation 2 (see Materials and methods), we found that 43 events reached a nominal significance level of 0.05 for comparison between cases and controls (Table 3). Five out of six randomly selected events that exhibited a *p *value less than 0.05 also were found to be significantly changed when subjected to more quantitative rtPCR analysis in a larger sample size (Table 2). Additionally, four events not identified as statistically significantly changed in the splice array screen (*p *> 0.05) were confirmed not to be changed using rtPCR.

**Table 3:** List of ion channel genes exhibiting alternative splicing in patients with Alzheimer's Disease

Genes by gene class	GenBank accession number (reference transcript*)	GenBank accession number (variant transcript*)	Alternative splicing event: change of mRNA transcript composition in AD^†^	Alternatively spliced region	Splice array *p *value
**Calcium channels**					
*CACNA1A*	NM_023035	CQ723237	ASA: 23% ↓ reference/total ratio	EXON 23	0.007
	NM_023035	U79668	ASD: 36% ↓ variant/reference ratio	EXON 48	0.022
*CACNA1C*	NM_000719	CQ722691	ASD: 19% ↑ reference/total ratio	EXON 27	0.033
*CACNA1G*	NM_018896	NM_198376	PIED: 14% ↓ variant/reference ratio	EXON 36	<0.001
*CACNA1I*	NM_001003406	AX068892	ASD: 29% ↓ variant/total ratio	EXON 16	0.049
*CACNA2D1*	NM_000722	BG210356	NE: 55% ↑ reference/total ratio	INTRON 3	0.021
					
**Chloride channels**					
*CLCN2*	NM_004366	BC021578	ES: 14% ↓ reference/total ratio	EXON 13	0.041
	NM_004366	BF002578	NE: 38% ↓ variant/total ratio	3' downstream	0.042
*CLCN6*	NM_001286	NM_021736	ES: 15% ↓ reference/total ratio	EXON 12	0.022
*CLCNKA*	NM_004070	CQ733615	ASD: 27% ↓ variant/reference	EXON 15	0.039
					
**Sodium channels**					
*SCN2A2*	NM_021007	CQ730725	ES: 16% ↓ variant/total ratio	EXON 11	0.023
*SCN7A*	NM_002976	BC062699	ES: 39% ↓ reference/total ratio	EXON 1	0.027
*SCNN1D*	NM_002978	AX230571	ASA: 17% ↓ variant/reference ratio	EXON 1	0.045
					
**Potassium channels**					
*KCNAB1*	NM_172160	NM_172159	NE: 21% ↓ variant/reference	INITRON 1	0.028
*KCNAB2*	NM_003636	AI933060	NE: 51% ↓ variant/total ratio	3' downstream	0.017
	NM_003636	AI090677	NEs: 105% ↑ variant/reference ratio	INTRON 7	0.039
	NM_003636	BM925038	PIED: 65% ↑ variant/reference ratio	EXON 16	0.045
*KCNF1*	NM_002236	AI566537	PIED: 51% ↑ variant/reference ratio	EXON 1	0.045
*KCNG1*	NM_172318	BF569197	ASD: 23% ↓ variant/reference ratio	EXON 1	0.005
	NM_172318	AI968477	ASD: 28% ↑ reference/total ratio	EXON 1	0.031
*KCNH5*	NM_172376	NM_139318	ES: 14% ↑ reference/total ratio	EXON 11	0.033
*KCNMB2*	NM_181361	NM_005832	NEs: 25% ↓ variant/total ratio	INTRON 1	0.030
*KCNN1*	NM_002248	BX106650	ASD: 47% ↑ reference/total ratio	EXON 6	0.004
	NM_002248	BM718136	ASD: 39% ↑ reference/total ratio	EXON 6	0.015
*KCNQ2*	NM_004518	NM_172107	NE: 70% ↑ variant/reference ratio	INTRON 10	0.038
	NM_004518	BG772772	NEs: 55% ↑ variant/reference ratio	EXONS 7-9	0.016
*KCNQ3*	NM_004519	CQ725469	PIED: 47% ↓ reference/total ratio	EXON 15	0.036
					
**GABA receptors**					
*GABRA3*	NM_000808	AX897950	ES: 48% ↑ reference/total ratio	EXON 7	0.037
*GABRA5*	NM_000810	AL035782	EsS: 65% ↑ variant/reference ratio	EXONS 2/3	0.030
*GABRA6*	NM_000811	AK090735	IR: 72% ↓ variant/total ratio	INTRON 6	0.037
					
**Ionotropic glutamate receptors**					
*GRIK2*	NM_021956	CQ715784	ASD: 57% ↓ reference/total ratio	EXON 10	0.007
*GRIK4*	NM_014619	CQ734018	NEs: 35% ↓ variant/total ratio	INTRON 1	0.006
*GRIK5*	NM_002088	AJ249209	NE: 52% ↑ variant/reference ratio	INTRON 18	0.037
					
**Transient receptor potential cation channels**					
*TRPM7*	NM_017672	CQ728707	ES: 6% ↓ reference/total ratio	EXON 4	0.048
					
**FXYD domain ion transport regulators**					
*FXYD5*	NM_014164	BU164524	NE: 36% ↓ variant/reference ratio	INTRON 1	0.030
					
**Inositol triphosphate receptors**					
*ITPR1*	NM_002222	CQ719499	ES: 15% ↓ reference/total ratio	EXON 23	0.030
					
**Two pore segment channels**					
*TPCN1*	NM_017901	BG899733	NE: 49% ↑ variant/reference ratio	INTRON 1	0.016
					
**Mucolipin**					
*MCOLN1*	NM_020533	CA489568	ASD: 95% ↑ variant/reference ratio	EXON 7	0.026
	NM_020533	AJ293659	IR: 33% ↑ variant/total ratio	INTRON 5	0.032
					
**Ryanodine receptors**					
*RYR1*	NM_000540	CQ717972	PIED: 30% ↑ reference/total ratio	EXON 91	0.011
	NM_000540	CQ730824	ES: 35% ↑ variant/reference ratio	EXON 35	0.042
					
**Miscellaneous other ion channels/interacting proteins**					
*TM4SF11 *(plasmolipin)	NM_015993	R16034	NE: 21% ↑ variant/reference ratio	INTRON 1	0.016
*GMRP-1 *(potassium channel tetramerization protein)	NM_032320	BG722430	NE: 26% ↑ variant/reference ratio	INTRON 1	0.031

*Reference transcript refers to traditionally splicing gene product, while the variant refers to the alternatively spliced mRNA transcript. ^†^Change was quantified using splice array samples (control versus AD temporal cortex, *n *= 10/group). The ratio selected to quantify event was determined based on best available probe choice (see Materials and methods section). ASA, alternative splice acceptor site; ASD, alternative splice donor site; ES, exon skipped; EsS, exons skipped; IR, intron retention; NE, novel exon; NEs, novel exons; PIED, partial internal exon deletion.

The splice array screen revealed 12% of ion channel genes in these experiments to be alternatively spliced in AD, with greater than 20% of genes comprising the calcium channel, chloride channel, sodium channel, and glutamate receptor gene class exhibiting AD-associated alternative transcript splicing. When compared to mTLE, there are fewer alternative splicing events in AD. Several explanations could account for this, with the simplest being that there are more alternative splicing events in mTLE compared to AD. On the other hand, the larger number of alternative splicing events in mTLE also could be explained by tissue differences between freshly resected and postmortem tissue. However, if this were the case and transcript degradation in the time prior to brain tissue collection were contributing to some of the observed splicing changes, we would expect to see a correlation between the splice variant proportions and time of brain tissue collection. This was not observed for any of the SVRs for splicing events identified in this study (data not shown). Another possible explanation might be that, in the AD cases, normalization of changes in the temporal cortex to those in the cerebellum reduces the baseline human-to-human variability in splicing proportions, thereby reducing the number of falsely identified events. While theoretically this could be the reason for the difference in the number of splicing events identified in mTLE and AD, caution should be used in accepting this interpretation. The comparison between AD and control is performed after two normalization steps, with the first being a probe normalization, followed by a brain region normalization. This results in an additional normalization step compared to the mTLE samples and, while reducing the subject-to-subject variability, the interassay variability is increased due to the compounding variability added with each ratio taken. It is possible that the extra normalization step results in more missed events due to an increase in variability. In fact, the *GRIA1 *(ionotropic (AMPA) glutamate receptor) gene was identified as a potential splice variant associated with AD in a preliminary study using the splice array technology (in *n *= 4 samples). In rtPCR this event was confirmed (Table 2) in a larger sample size. However, when additional samples were studied by splice array analysis, this event was no longer detected as being changed in the complete sample set (*n *= 10) employed in this arm of the study. This also occurred for the alternative splicing event of the *KCCN2 *gene in AD. This strongly suggests that our list of events is a minimum estimate of the ion channel splice events that are associated with AD. However, in order to minimize the number of false positives we report due to human-to-human variability (in most cases considered to be greater than interassay variability), we applied a conservative data analysis approach given the tissue samples available to us for these studies.

**Figure 2:**
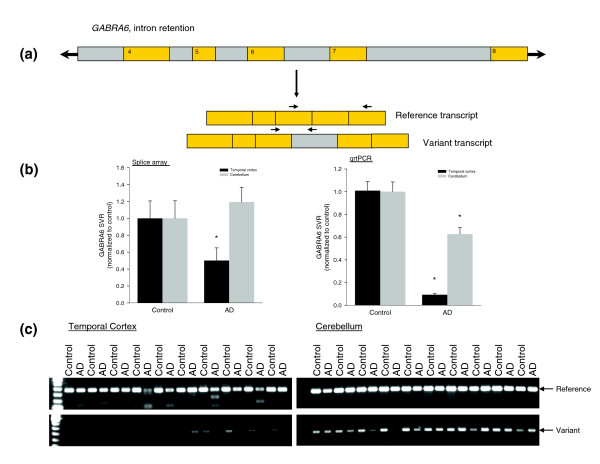
Representative AD-associated alternative splicing event identified using splice array technology. **(a) **Schematic of *GABRA6 *alternative splicing event associated with mTLE. Exons are shown in orange and intronic regions are shown in gray. **(b) **Data collected for the *GABRA6 *alternative splice event in control and AD brain tissue (TC and CB) samples using the splice array technology (left) and quantitative rtPCR (qrtPCR, right). Data are presented as mean ± standard error of the mean; *p < 0.05 when compared to control, Student's *t*-test. **(c) **rtPCR confirmation of the pattern of transcript expression in brain tissue collected from ten subjects from each group. Reference and variant transcript forms were amplified using the following primer sequences (indicated in the figure by arrows above mRNA transcripts): reference GABRA6F-AAGAATCTTCAAGCCTTCTCCA, GABRA6R-TGACAGCTGCGAACTCGATA, variant GABRA6F-AAGAATCTTCAAGCCTTCTCCA, GABRA6F-TCCAAGATTACACAAATCTTTATATGC.

A representative AD-associated alternative splicing event is shown in Figure 2. This selected event in the *GABRA6 *gene results in the inclusion of an intronic sequence located between exons 6 and 7 (Figure 2a). Both the splice array data and the rtPCR confirmation revealed a reduction in the variant form containing the intronic sequence in AD brain tissue samples (Figure 2b). In the splice array evaluation of this event, a statistically significant reduction was observed in temporal cortex from AD subjects compared to controls, while no such change was seen in the cerebellar samples. Quantitative rtPCR revealed, however, a statistically significant AD-associated reduction in the variant form in both the temporal cortex and cerebellum, with a much larger change detected in the temporal cortex. Given the enhanced sensitivity of rtPCR, this difference is not unexpected and likely results from rtPCR picking up on a more subtle change in the cerebellum. Reverse transcriptase amplification of the transcript variants in selected brain tissue samples reveals the effects of the disease state on reducing the variant splice form of *GABRA6*, with a greater magnitude of change in the transcripts containing the intronic region detected in the disease-affected brain tissue structure (temporal cortex, Figure 2c).

## Discussion

Bioinformatic investigations have permitted the comprehensive compilation of expressed sequence tag (EST) and cDNA databases that identify potential sites of alternative splicing in human genes. The technological capability now exists to discover the extent to which splicing changes are present in human disease. Using mTLE and AD as prototypical neurological diseases, we specifically sought to identify alternative splice variants of ion channel genes in human brain tissue samples to address the following goals: to compile a list of ion channel genes in mTLE and AD that undergo alternative splicing; and to validate the use of new technology in the evaluation of ion channel splice variation for a large set of genes in a high-throughput manner. Based on these results, we propose a strategy for selection of the most clinically relevant splicing changes to be pursued in further functional and mechanistic studies.

### Disease-associated alternative ion channel splicing

#### Alternative splicing of ion channels in mTLE

The splice array technology identified widespread alternative splicing changes associated with mTLE. Importantly, the well-characterized mTLE-associated flip-flop alternative splicing events of the ionotropic glutamate receptor (splicing change conserved across all neuronally expressed *GRIA *genes) were detected in both *GRIA1 *and *GRIA2 *genes in these experiments (Table 1). Our results are in agreement with the previously published finding of an upregulation of the flip splice form in mTLE [19,20].

We also have demonstrated splicing changes in many genes with known associations to human epilepsies. Specifically, we have identified two mTLE-associated splicing events (Table 1) in the *KCNQ2 *gene, which encodes a voltage-gated potassium channel. This gene is of particular interest in epilepsy as several mutations in it have been associated with benign familial neonatal convulsions [11,21-26]. Furthermore, many of the mutations have been located in splice sites surrounding exons [23,24,26], possibly implicating them in alternative splicing events identified herein. Several other genes that we have identified as undergoing mTLE-linked alternative splicing events also have been associated with other, non-mTLE forms of human epilepsy, including *CACNA1A*, *CACNA1H*, *CLCN2*, and *GABRG2 *[27-30], indicating that these also may be of particular interest in the epilepsy field.

We emphasize that in screening for alternative splicing events associated with mTLE, we employed neocortical brain tissue. While the hippocampus is generally felt to be the disease focus in this form of epilepsy, the hippocampus is also the site of greatest tissue damage. The intent of this work was to investigate a high-throughput method of splice variant identification. Therefore, to reduce potential confounding issues of significant non-uniform neuronal loss and/or altered cellular composition, we pursued changes in the temporal neocortex, adjacent to the diseased hippocampus, rather than in the hippocampus itself. We believe that this approach minimizes the number of artifactual splicing changes. Now that a list of potential TLE-linked splicing changes has been identified, additional work is needed to evaluate the extent of these changes in the hippocampus. To do this it will be necessary to use laser capture microdissection to separate intact neurons in the hippocampus to assess splice variation in homogeneous cell populations in this brain structure. Events that occur more extensively in the hippocampus compared to the neocortical tissue may indicate a more direct role in disease pathology.

#### Alternative splicing of ion channels in AD

AD also revealed extensive alternative ion channel splicing. No information has been reported to date regarding alternative splicing of ion channel genes associated with AD, although several ion channels that we have identified as undergoing alternative splicing have been implicated in AD. Specifically, our data show a splicing change in *GRIA1 *in AD (Table 3). *GRIA1 *encodes an ionotropic (AMPA) glutamate receptor, one of a class of genes that have been extensively linked to cognition and memory [31]. A downregulation of *GRIA1 *expression has been observed in diseased brain regions collected from subjects with AD [14,17,18]. Furthermore, mice deficient in GluR-A (encoded by *gria1*) have been shown to have deficits in long-term potentiation and reductions in spatial working memory tasks [32-34]. Therefore, alternative splicing of the *GRIA1 *gene may have a pathophysiological role in AD. Based on previous literature, the splicing change identified in *ITPR1 *(Table 3) may also play an important role in AD. Presenilin, a transmembrane protein localized to endoplasmic reticulum, is required for the proteolysis of amyloid precursor protein. Evidence suggests that mutations in the presenilin gene causing early onset AD [35] may result in alterations in inositol-triphosphate signaling (via *ITPR *receptors) to increase intracellular calcium [36]. In addition to alternative splicing in *ITPR1*, our data also show several alternative splicing events in calcium channel genes that also may factor into the pathway of intracellular calcium dysregulation commonly associated with AD [15].

### Validation of disease-associated alternative splicing

The splice array technology has proven valuable for comprehensively identifying splice variation across a large set of genes. The accuracy of the splice array technology is evident by the reproducibility of the results using more quantitative rtPCR. While events could have been missed using our data analysis strategy, the reported list likely reflects few false positive results given the follow-up rtPCR success rate and the reproducibility in a relatively large sample size. Collectively evaluating all of our rtPCR confirmation studies, we estimate that >80% of all array-identified splicing changes (Tables 1 and 3) are real events.

All splicing events identified in this work exhibit only small magnitude changes, with the maximum exceeding just over a 2.5-fold change. Despite the relatively low magnitude of changes, if these transcripts encode proteins with altered function then the biological consequences could be substantial. The fact that previously identified splicing changes in the *GRIA *genes, which have been well-established in the literature to be involved in both human epilepsy and animal models [19,20,37-40], were observed in mTLE brain tissue at only a 30% increase suggests that these low level splicing changes could be real and biologically important alternative splicing events.

**Figure 3:**
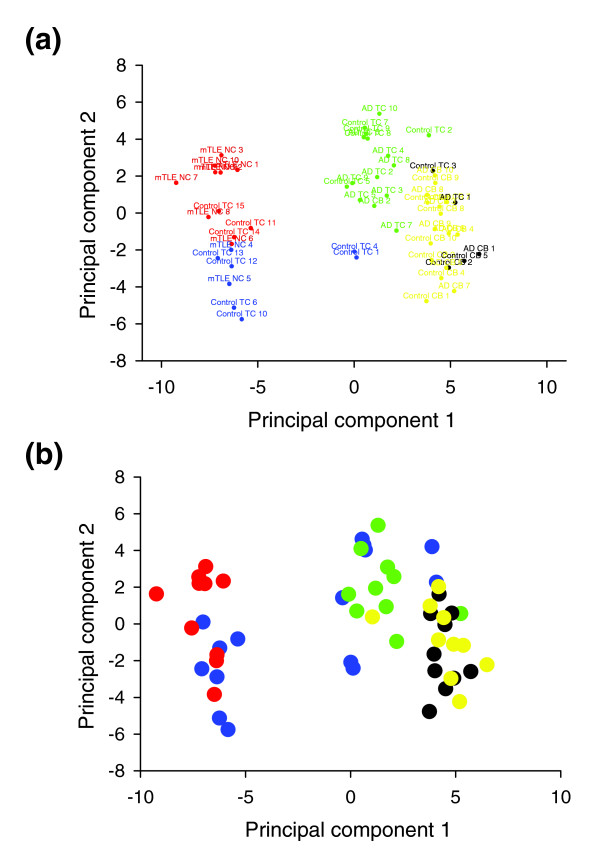
Principal component analysis of ion channel splice variant expression patterns for all experimental groups. **(a) **Colors separate groups based on statistical clustering (k-means clustering) of individuals with similar patterns of ion channel splicing. **(b) **Colors separate groups based on disease state and brain structure: control TC (blue), mTLE NC (red), AD TC (green), control CB (black), AD CB (yellow). The first principal component explains 43% of the variation in log expression ratios, while the second principal component accounts for 13% of the variation. Separation of clusters along principal components 1 and 2 is, to a large extent, governed by brain structure and disease state.

To provide further validation of the accuracy of the technology in identifying true alternative splicing differences in the experimental groups, we employed principal component and k-means clustering analyses to collectively evaluate patterns of splicing changes in the brain tissue samples. Regression analysis of the first two principal components (PCs; Figure 3) indicates that the largest sources of variability are accounted for by brain structures (temporal cortex or cerebellum) and by disease state. Across PC1 and PC2 combined, 37% of variation is explained by membership of the epilepsy (mTLE NC) group. Outside of epilepsy samples, 30% is explained by differences in brain tissue type, and an additional 11% is explained by differences between AD and control samples (combining main and interaction effects with tissue type). We note that while the majority of the control temporal cortex (Control TC) samples cluster together, these samples have several outliers from the dominant cluster (blue cluster, Figure 3). This is likely due to the lack of homogeneity of these patients with regard to disease states, past medical history, and current medications at the time of death. In fact, with regard to the medical history of the controls, all that is known is that they did not have epilepsy and had minimal cognitive dysfunction. Despite the lack of precise grouping in the controls, the mTLE and AD subjects cluster together well and, in both conditions, the groups do diverge from the dominant control subject cluster. Importantly, the clustering analyses place the control, AD, and mTLE temporal cortex results predominantly in unique groups, while the control and AD cerebellar samples are largely grouped together (Figure 3a). Given that the cerebellum in AD patients is considered a largely unaffected brain structure, the overlap of control and AD cerebellum results is not unexpected and validates the use of this structure as an internal control for improving the ability to detect disease related changes in the temporal cortex. Furthermore, for AD it was possible to distinguish the contribution of disease state in both the temporal cortex (affected in AD) and cerebellum (largely unaffected in AD) using linear regression modeling.

Using this approach, we determined that, in the temporal cortex, 20% of the variability accounted for by PC1 and PC2 is attributed to disease state, whereas in the cerebellum only 0.4% of the variability could be accounted for by AD-status. Taken together, these analyses provide strong validation for the accuracy of the splice array in detecting real alternative splicing changes in the disease states.

#### Effects of altered cellular composition in disease states

Both mTLE and AD are diseases that result in neurodegeneration in patients to varying degrees between affected individuals and between brain regions. A concern arises that the alternative splicing changes we are observing in this work are due to alteration in cellular composition. To address this issue, we undertook immunoblot analysis to assess neuronal and glial cell numbers. Immunoblot results were obtained for control (*n *= 24), mTLE (*n *= 22), and AD (*n *= 31) temporal cortex. Samples that did not have adequate protein amount to evaluate their NeuN (neuronal nuclei, neuronal marker) and GFAP (glial fibrillary acidic protein, glial cell marker) amounts accurately were excluded from the analysis. Despite the ability to detect group-specific changes in cellular populations (Additional data figure 1 in Additional data file 2), neither the splice array-determined splice proportions, nor data collected using rtPCR, showed any statistically significant correlation to the NeuN or GFAP protein content. A representative graph showing this lack of correlation is given in Additional data figure 2 (in Additional data file 2) for *MCOLN1 *SVR in control, mTLE, and AD temporal cortical tissue. All other events demonstrated a similar lack of correlation between SVRs and NeuN or GFAP protein concentration. Although immunoblotting might not be quantitative enough to detect slight changes in cellular composition that likely are occurring in these brain tissue samples, given that we were able to detect quantitative differences between experimental groups (Additional data figure 1 in Additional data file 2) consistent with neurodegeneration (Additional data file 1), we believe that alterations in cellular composition are not the major cause of the observed disease-associated splicing changes.

### Functional consequences of alternative splicing

#### Splice variants with known function

We cannot determine if particular splice variant transcripts identified in this work are translated into proteins with altered function, if translation does not occur for a splice variant, or if a resulting protein is immediately degraded because of dysfunctional activity. Theoretically, more drastic changes could result from a translated protein with altered function, otherwise alteration would simply be a low level expression change. Few splicing events detected by the splice array have information about the presence of an associated protein and even fewer have functional data available for the proteins translated from the splice variant transcripts. Of all events identified in our work, only *CACNB4 *(GenBank: AY054985; Tables 1 and 2) and the previously reported *GRIA *gene splicing changes (flip-flop), identified to be present in increased quantities in mTLE patients, have data regarding the functional consequences of the splice variant [37,41].

*CACNB4 *encodes the β subunit of calcium channels and modulates the activity of the pore-forming α subunits [42]. The function of the protein produced from the splice variant compared to the normal transcript was studied in *Xenopus *oocytes in the presence of α subunits. The protein encoded by the splice variant inactivated at a faster rate and the voltage dependence of the α/β complex was shifted to more depolarized potentials in the presence of the splice variant of *CACNB4 *[41]. Thus, in the presence of this splice variant, the neuron may be more likely to depolarize with a smaller stimulus. The *GRIA *gene splice variants also have been shown to exhibit electrophysiological properties consistent with enhanced cellular excitability [37]. While the direct biological consequences of the *CACNB4 *and *GRIA *gene splicing changes in patients with mTLE cannot be concluded from this work, the observation of expressed proteins with altered function is important and warrants follow-up investigations to decipher how the changes may contribute to mTLE. Specifically, for both *CACNB4 *and *GRIA *splicing changes, it would be interesting to evaluate the relationship between SVRs and seizure frequency/severity in animal models of epilepsy to assess *in vivo *the consequences of the alterations.

#### Elucidating functional consequences of splice variants with unknown function

This work has, for the first time, established specific disease-associated alternative splicing events across a broad category of genes in two neurological diseases. With the generation of these lists of disease-associated ion channel alternative splicing events comes the task of deciphering the functional consequences of these changes and how they may contribute to the diseases. There are too many splicing changes associated with mTLE and AD to pursue comprehensive functional evaluation to determine the biological impact of all proteins expressed from alternatively spliced mRNA transcripts. A strategy is clearly needed for selection of events for functional evaluations.

One approach to identify the most clinically significant alternative splicing events is to evaluate the compiled lists for mTLE (Table 1) and AD (Table 3) for splicing events that are genetically controlled. Specifically, we propose evaluating splice donor and acceptor regions for single nucleotide polymorphisms that may be responsible for alterations in SVRs and to evaluate correlations between SVRs and genotypes. We can then evaluate a large population of affected individuals for the presence of the polymorphism and certain clinical phenotypes, such as measures of disease severity or responsiveness to medications. In doing this, we would be able to indirectly associate the splicing event with key clinical outcomes. This approach would focus mechanistic investigation upon only the most disease-relevant changes. Furthermore, in knowing that a splicing change contributes to a specific clinical endpoint, functional investigations can be designed to evaluate specific and relevant mechanistic hypotheses. This approach is exemplified by an *SCN1A *polymorphism located in the 5' consensus site region following the neonatal form of exon 5 that has been found to be a genetically controlled key alternative splicing event [43]. This polymorphism has been associated with altered doses of certain antiepileptic drugs in two independent populations [6,44]. These findings have led to the hypothesis that the pharmacology of these antiepileptic drugs may be altered in the presence of decreased expression of the splice variant, and have guided follow-up mechanistic work into how this might be occurring. While many of the identified splicing changes reported herein are not genetically controlled, it is not unreasonable to expect that a proportion of them are. In cases where it is possible, establishing genetic control of alternative splicing will serve as a valuable platform for beginning to decipher their disease significance.

## Conclusion

Based on the few known examples of disease-associated splicing events, the impact of elucidating the contribution of alternative transcript forms in human disease likely will be substantial. EST and cDNA libraries provide valuable information about potential alternative splice forms on a genome-wide scale. It is not currently feasible to determine in a high throughput manner which of these numerous events result in a change in system biology. One possible direction is to concentrate on the functional significance of variant transcripts associated with human disease. We undertook such a task by evaluating brain tissue from mTLE and AD for disease-associated alternative splice variants using a splice variant microarray, the first reported example of employing such an approach in neurological disease. Collectively, our results demonstrate that two common and important neurological conditions are associated with widespread alterations in splicing patterns of ion channel genes. While additional experimentation is needed to establish the consequences of these alternative splicing events in the diseases, these lists provide a valuable foundation for elucidating which of these events translate into clinically significant changes.

## Materials and methods

### Brain tissue samples

This study was approved by the Joint Research Ethics Committee of the Institute of Neurology and the National Hospital for Neurology and Neurosurgery, and the Duke University Institutional Review Board.

Freshly resected brain tissue from patients with mTLE was used to identify key splicing changes associated with this type of epilepsy. All these patients gave written informed consent for use of the resected brain tissue for research. Temporal neocortical tissue samples (*n *= 43) were obtained from material resected from patients undergoing therapeutic surgery for drug-resistant mTLE according to routine clinical protocols at the National Hospital for Neurology and Neurosurgery (London, UK). All tissue used for research was surplus to diagnostic requirements. Patients were between the ages of 18 and 60 years and the group consisted of 15 males and 28 females. All had a diagnosis of hippocampal sclerosis confirmed histologically. The hippocampal tissue was excluded from analysis due to potential confounding issues of extensive, non-uniform, neuronal loss.

Nonepileptic/nondemented autopsy control and AD temporal cortical and cerebellar tissue was obtained through the Kathleen Price Bryan Alzheimer's Disease Brain Bank at Duke University. Subjects were enrolled prospectively and followed until death as previously described [45]. Temporal cortex samples were tested as this area is highly affected by AD, while the cerebellum has minimal AD pathology, and was, therefore, used as an internal control. Control subjects (*n *= 31) were between the ages of 56 and 90 years (mean 85.5 years) and consisted of 14 males and 17 females. AD patients (*n *= 32) were between the ages of 79 and 90 years (mean 83.5 years) and consisted of 8 males and 24 females. All AD subjects had AD pathology greater than Braak stage IV. All brain tissue was collected postmortem with the time to collection ranging from 1-30 h (mean 9.5 h) and 0.5-18.5 h (mean 8 h) for control and AD, respectively. Autopsy control samples were used as the control group for both epilepsy and AD. All brain tissue was flash frozen in liquid nitrogen and stored at -80°C until use.

### Splice array

Ion channel splice arrays were purchased through ExonHit Therapeutics (Gaithersburg, MD, USA). All known and postulated alternative splicing events involving ion channel genes and their major associated interacting proteins were included on the array (1,665 events in total). Each alternatively spliced transcript defined a splicing event. The reference transcript was chosen for each gene by the company as one of the most commonly accepted mRNA transcript forms. The splice variant for a particular event is referred to as the variant transcript. In all cases where a splicing event was identified to be altered in epilepsy or AD, the GenBank accession numbers for both transcript forms and the affected exons are provided in Tables 1 and 3 to define the specific exon structure for the gene undergoing alternative splicing.

For each splicing event analyzed on the splice array, three to six probes were used to quantify the event. The number of probes selected for an event depended on the nature of the splicing event and the ability to design an adequate probe for the desired sequence (more detail about probe design is given in Fehlbaum *et al*. [8]). Specifically, the complete probe sets consisted of an A3 probe to monitor total transcript expression at the 3' end, an A5 probe for assessing total transcript expression at the 5' end, a B probe designed to bind to a purely exonic region specific to the reference or the variant (which transcript depends on the event), a C and D probe to screen for exon-exon junctions surrounding an exon unique to the reference or the variant, and an E probe to assess a unique exon-exon boundary in the transcript form not being screened for by the B probe.

### RNA extraction and splice array data collection

Brain tissue was ground in liquid nitrogen (mortar and pestle). Tissue powder was separated into a 20-30 mg fraction for RNA extraction and a 5-10 mg sample for protein extraction. Total RNA was extracted from brain tissue using the RNeasy Lipid Tissue Purification Kit (Qiagen, Valencia, CA, USA) according to the manufacturer's instructions. The resulting RNA was quantified spectrophotometrically. RNA was extracted from all subjects comprising the experimental groups (*n *= 43 for mTLE, *n *= 29 for control temporal cortex (mTLE), *n *= 32 AD temporal cortex, and *n *= 31 control (AD)). RNA from a subset of ten subjects from each group was selected for splice array analysis.

Samples were selected for splice array analysis to provide for the closest possible age and gender comparisons. To do this effectively for each disease to control comparison, five of the ten control samples overlapped for the AD and epilepsy evaluations. Coded aliquots of extracted total RNA from the selected subjects were sent to ExonHit Therapeutics for Ion Channel splice array analysis. Prior to dye-labeling and hybridization, the RNA quality was assessed on an Agilent Bioanalyzer (Palo Alto, CA, USA).

For the epilepsy splice variation scan, a set of ten samples per group (controls and epilepsy samples) were submitted for the comparison between mTLE neocortex and control temporal cortex (control temporal cortex 1). A mTLE neocortex and a control temporal cortex 1 sample were labeled with cy3 and cy5 fluorescent dyes and hybridized to the splice array chip simultaneously (two samples labeled with different dyes per array). A total of ten splice arrays were used for the disease to control comparison. The effects of potential dye bias were minimized by labeling five of the ten samples comprising a group with opposite dyes.

For the AD splice variation scan, splice array data were generated for a set of ten samples per group (AD temporal cortex, control temporal cortex 2, cerebellum AD, and cerebellum control 2). In all cases, matched temporal cortical and cerebellar tissue samples were obtained from each subject. An AD and control sample were run together on a single chip in an identical manner as described for the mTLE samples. The same control versus AD comparison also was made for the cerebellar samples. A total of 20 arrays were used for the analysis, with 10 arrays used for temporal cortex comparisons and 10 arrays used for cerebellar analyses.

All splice array data files are available through the Gene Expression Omnibus (series accession numbers: mTLE neocortex (GSE6773), control temporal cortex 1 (GSE6771), AD temporal cortex (GSE6775), control temporal cortex 2 (GSE6774), cerebellum AD (GSE6777), and cerebellum control 2 (GSE6778)).

### Splice array data analysis

In some cases, probes included on the splice array do not hybridize to the RNA in the experimental sample either due to a lack of the targeted sequence in the sample or because of a failure of the probe to bind in a sample type. To address this issue, probe hybridization fluorescence output values were excluded for a particular splicing event if values were <200, as this approaches the limit of detection of this technology. A single probe hybridization value of <200 in a group resulted in the complete elimination of that probe from the quantification of that splice variant for that control versus disease comparison. Probe sets were eliminated across the entire disease-control comparison only if they failed to hybridize in a particular brain structure regardless of disease state, and not if they failed solely in the control or diseased brain tissue structure, as the latter could be caused by a disease-altered splicing change.

Ratios of probe hybridization fluorescence outputs were used to quantify the relative number of alternatively spliced transcripts, and to control for any alterations in overall gene expression. Selection of the best possible ratio for an event was based on the following ratio priority: E/B > B/A3 = B/A5 > E/A3 = E/A5 > E/C = E/D. This priority system was largely based on the rationale that the most sensitive measure of an alternative splicing event will be to directly quantify variant to reference ratios. This is particularly the case when the changes in the reference and variant transcripts occur in concert (that is, a reduction in reference occurs simultaneously with an increase in variant). However, junction probes generally are shorter in length and, therefore, emit a less reliable fluorescence output. Thus, for this study we accepted the less reliable junction probe when it was normalized to a more reliable nonjunction probe (E/B), but deemed the dual junction probe ratio (E/C or E/D) to be of lower priority. The A3 and A5 probes are longer probes that often span several exons and, therefore, provide reliable hybridization fluorescence outputs. However, in a gene where there are several alternative splicing events, normalizing to one of the probes screening for total transcript expression (A3 or A5) may actually be quantifying more than one splicing event. Therefore, the strategy of normalizing to total transcript expression was placed as lower priority to the more specific variant/reference (E/B) normalization ratio. In addition to this priority system, it also was necessary to select a ratio that was available in all groups for most accurate comparison. For example, when comparing AD to control, the E/B ratio would be selected only if it were available in the AD and control temporal cortex and cerebellum samples. If the E/B ratio was not available for any of the groups (due to one of the probes being below the limit of detection in a group), then the next highest priority ratio was selected. The selected ratio was defined as the SVR for a particular variant.

Using this selection procedure, SVRs were defined for all of the splice variants for both the mTLE and AD comparisons. For AD, following selection of a common SVR across all four groups, an additional ratio was taken normalizing the temporal cortex ratio to the cerebellum ratio (TC/CB) for each individual. A Student's *t*-test was then used to identify particular splicing events with a statistically significant change in epilepsy and AD using the following comparisons:

*mTLE: SVR*_*TC*, *control*2 _*vs*. *SVR*_*NC*, *mTLE *_    (equation 1)

$AD:\frac{SVR_{TC,control1}}{SVR_{CB,control1}}\;vs.\;\frac{SVR_{TC,AD}}{SVR_{CB,AD}}\;\left(\text{equation }2\right)$

Lists were made of the events that had a statistically significant alteration (*p *< 0.05). Of these, several were selected for quantitative real time PCR follow-up evaluations to test the accuracy of the splice array findings. As negative controls, a total of four events that were not found to be significantly changed in mTLE or AD were also selected for follow-up rtPCR confirmation. rtPCR follow-up was carried out in a larger sample set, also including all of the original samples.

All splice array data analysis was performed in Microsoft Excel. All data inputs and calculations were verified by two individuals. Final compiled sheets were checked by manual calculation of randomly selected splicing events.

Due to the differences in time of brain tissue collection in the AD experiments, linear regression analyses were performed comparing the proportion of alternative transcripts versus the time of postmortem brain tissue collection in control and AD brain tissue. For events identified in epilepsy, correlations were assessed only in control tissue, as all the mTLE neocortical tissue was freshly collected.

### Real time PCR

To confirm splicing events identified in the splice array, and to estimate the magnitude of the effect in a larger set of samples, rtPCR was employed. RNA from all subjects comprising the groups (including the ten sent for splice array analysis) was used in the rtPCR analyses. A total RNA sample from all subjects was reverse transcribed into cDNA using a High Capacity cDNA Synthesis Kit (Applied Biosystems, Foster City, CA, USA) according to product instructions. Taqman^® ^assays and custom-designed primer/probe sets that were used in rtPCR studies to confirm the presence of the splice variants are given in Additional data file 1. All rtPCR assays were purchased through Applied Biosystems and run per the manufacturer's protocol using 10-20 ng of total RNA (converted to cDNA) per reaction.

Standard curves were run for a range of total RNA (converted to cDNA, 9 standards ranging from 0.01-100 ng). Number of cycles to reach a threshold fluorescence reading (C_T_) was plotted against log RNA amount and a line was fitted to the data points using linear regression. The following equation was used to quantify the number of transcripts present in an experimental sample:

Number of transcripts=10^((CT,Sample−y0)α)     (equation 3)

Where, C_T, sample _is the number of cycles to reach the fluorescence threshold for a given sample, and y_0 _and α are, respectively, the intercept and slope of the line defining the relationship of C_T _versus log_(RNA amount) _determined from the standard curve [46]. Using this approach, differences in amplification efficiency are accounted for, thereby permitting relative quantification between the two transcript forms. Relative proportions of splice variants were quantified by dividing the number of transcripts in the variant form to the number of transcripts in the reference form. Occasionally, it was necessary to normalize to total expression of the two transcripts forms (by probing for transcript region common to both) due to assay development restrictions.

### Principal components analyses

Principal component analyses on covariances between log-transformed SVRs within individuals were used to identify the most significant sources of variability between brain tissue samples collected from diseased and nondiseased patients. K-means clustering was performed to group individuals with similar alternative splicing profiles. Linear regression models were fitted to the data points defining AD and control samples along the most important principal component axes to determine the contribution of disease state in both disease-affected and unaffected brain structures. Principal component analyses were performed using JMP software (version 5.1.2, Cary, NC, USA). Linear regression analyses were performed using SPSS software (version 13, Chicago, IL, USA).

## Additional data files

The following additional data are available with the online version of this paper. Additional data file 1 contains Taqman^® ^assays and custom-designed primer/probe sequences that were used in rtPCR studies to confirm the presence of the splice variants. Additional data file 2 contains additional methods, discussion and figures regarding the assessment of cellular composition of the brain tissue samples used in these studies.

## Supplementary Material

Additional data file 1 Taqman^® ^assays and custom-designed primer/probe sequences used in rtPCR studies to confirm the presence of the splice variants. Click here for file

Additional data file 2 Additional methods, discussion and figures regarding the assessment of cellular composition of the brain tissue samples used in these studies. Click here for file

## References

[B1] Syken J De-Medina T Munger K TID1, a human homolog of the *Drosophila *tumor suppressor l(2)tid, encodes two mitochondrial modulators of apoptosis with opposing functions. Proc Natl Acad Sci USA 1999 96 8499 8504 10411904 10.1073/pnas.96.15.8499 PMC17545

[B2] Cartegni L Krainer AR Disruption of an SF2/ASF-dependent exonic splicing enhancer in SMN2 causes spinal muscular atrophy in the absence of SMN1. Nat Genet 2002 30 377 384 10.1038/ng854 11925564

[B3] Hutton M Lendon CL Rizzu P Baker M Froelich S Houlden H Pickering-Brown S Chakraverty S Isaacs A Grover A Association of missense and 5'-splice-site mutations in tau with the inherited dementia FTDP-17. Nature 1998 393 702 705 10.1038/31508 9641683

[B4] Glatz DC Rujescu D Tang Y Berendt FJ Hartmann AM Faltraco F Rosenberg C Hulette C Jellinger K Hampel H The alternative splicing of tau exon 10 and its regulatory proteins CLK2 and TRA2-BETA1 changes in sporadic Alzheimer's disease. J Neurochem 2006 96 635 644 10.1111/j.1471-4159.2005.03552.x 16371011

[B5] Faustino NA Cooper TA Pre-mRNA splicing and human disease. Genes Dev 2003 17 419 437 10.1101/gad.1048803 12600935

[B6] Tate SK Depondt C Sisodiya SM Cavalleri GL Schorge S Soranzo N Thom M Sen A Shorvon SD Sander JW Genetic predictors of the maximum doses patients receive during clinical use of the anti-epileptic drugs carbamazepine and phenytoin. Proc Natl Acad Sci USA 2005 102 5507 5512 15805193 10.1073/pnas.0407346102 PMC556232

[B7] Clark TA Sugnet CW Ares M Jr Genomewide analysis of mRNA processing in yeast using splicing-specific microarrays. Science 2002 296 907 910 10.1126/science.1069415 11988574

[B8] Fehlbaum P Guihal C Bracco L Cochet O A microarray configuration to quantify expression levels and relative abundance of splice variants. Nucleic Acids Res 2005 33 e47 15760843 10.1093/nar/gni047 PMC1064144

[B9] Johnson JM Castle J Garrett-Engele P Kan Z Loerch PM Armour CD Santos R Schadt EE Stoughton R Shoemaker DD Genome-wide survey of human alternative pre-mRNA splicing with exon junction microarrays. Science 2003 302 2141 2144 10.1126/science.1090100 14684825

[B10] Pan Q Saltzman AL Kim YK Misquitta C Shai O Maquat LE Frey BJ Blencowe BJ Quantitative microarray profiling provides evidence against widespread coupling of alternative splicing with nonsense-mediated mRNA decay to control gene expression. Genes Dev 2006 20 153 158 16418482 10.1101/gad.1382806 PMC1356107

[B11] Moulard B Picard F le Hellard S Agulhon C Weiland S Favre I Bertrand S Malafosse A Bertrand D Ion channel variation causes epilepsies. Brain Res Brain Res Rev 2001 36 275 284 10.1016/S0165-0173(01)00104-7 11690625

[B12] Hynd MR Scott HL Dodd PR Glutamate-mediated excitotoxicity and neurodegeneration in Alzheimer's disease. Neurochem Int 2004 45 583 595 10.1016/j.neuint.2004.03.007 15234100

[B13] Angulo E Noe V Casado V Mallol J Gomez-Isla T Lluis C Ferrer I Ciudad CJ Franco R Up-regulation of the Kv3.4 potassium channel subunit in early stages of Alzheimer's disease. J Neurochem 2004 91 547 557 10.1111/j.1471-4159.2004.02771.x 15485486

[B14] Dewar D Chalmers DT Graham DI McCulloch J Glutamate metabotropic and AMPA binding sites are reduced in Alzheimer's disease: an autoradiographic study of the hippocampus. Brain Res 1991 553 58 64 10.1016/0006-8993(91)90230-S 1933277

[B15] LaFerla FM Calcium dyshomeostasis and intracellular signalling in Alzheimer's disease. Nat Rev Neurosci 2002 3 862 872 10.1038/nrn960 12415294

[B16] Oddo S LaFerla FM The role of nicotinic acetylcholine receptors in Alzheimer's disease. J Physiol Paris 2006 99 172 179 10.1016/j.jphysparis.2005.12.080 16448808

[B17] Wakabayashi K Narisawa-Saito M Iwakura Y Arai T Ikeda K Takahashi H Nawa H Phenotypic down-regulation of glutamate receptor subunit GluR1 in Alzheimer's disease. Neurobiol Aging 1999 20 287 295 10.1016/S0197-4580(99)00035-4 10588576

[B18] Yasuda RP Ikonomovic MD Sheffield R Rubin RT Wolfe BB Armstrong DM Reduction of AMPA-selective glutamate receptor subunits in the entorhinal cortex of patients with Alzheimer's disease pathology: a biochemical study. Brain Res 1995 678 161 167 10.1016/0006-8993(95)00178-S 7542540

[B19] Seifert G Schroder W Hinterkeuser S Schumacher T Schramm J Steinhauser C Changes in flip/flop splicing of astroglial AMPA receptors in human temporal lobe epilepsy. Epilepsia 2002 43 Suppl 5 162 167 10.1046/j.1528-1157.43.s.5.10.x 12121314

[B20] de Lanerolle NC Eid T von Campe G Kovacs I Spencer DD Brines M Glutamate receptor subunits GluR1 and GluR2/3 distribution shows reorganization in the human epileptogenic hippocampus. Eur J Neurosci 1998 10 1687 1703 10.1046/j.1460-9568.1998.00171.x 9751141

[B21] Tang B Li H Xia K Jiang H Pan Q Shen L Long Z Zhao G Cai F A novel mutation in KCNQ2 gene causes benign familial neonatal convulsions in a Chinese family. J Neurol Sci 2004 221 31 34 10.1016/j.jns.2004.03.001 15178210

[B22] Singh NA Westenskow P Charlier C Pappas C Leslie J Dillon J Anderson VE Sanguinetti MC Leppert MF BFNC Physician Consortium KCNQ2 and KCNQ3 potassium channel genes in benign familial neonatal convulsions: expansion of the functional and mutation spectrum. Brain 2003 126 2726 2737 10.1093/brain/awg286 14534157

[B23] Singh NA Charlier C Stauffer D DuPont BR Leach RJ Melis R Ronen GM Bjerre I Quattlebaum T Murphy JV A novel potassium channel gene, KCNQ2, is mutated in an inherited epilepsy of newborns. Nat Genet 1998 18 25 29 10.1038/ng0198-25 9425895

[B24] Lee WL Biervert C Hallmann K Tay A Dean JC Steinlein OK A KCNQ2 splice site mutation causing benign neonatal convulsions in a Scottish family. Neuropediatrics 2000 31 9 12 10.1055/s-2000-15290 10774989

[B25] de Haan GJ Pinto D Carton D Bader A Witte J Peters E van Erp G Vandereyken W Boezeman E Wapenaar MC A novel splicing mutation in KCNQ2 in a multigenerational family with BFNC followed for 25 years. Epilepsia 2006 47 851 859 10.1111/j.1528-1167.2006.00552.x 16686649

[B26] Claes LR Ceulemans B Audenaert D Deprez L Jansen A Hasaerts D Weckx S Claeys KG Del-Favero J Van Broeckhoven C *De novo *KCNQ2 mutations in patients with benign neonatal seizures. Neurology 2004 63 2155 2158 15596769 10.1212/01.wnl.0000145629.94338.89

[B27] Kananura C Haug K Sander T Runge U Gu W Hallmann K Rebstock J Heils A Steinlein OK A splice-site mutation in GABRG2 associated with childhood absence epilepsy and febrile convulsions. Arch Neurol 2002 59 1137 1141 10.1001/archneur.59.7.1137 12117362

[B28] Haug K Warnstedt M Alekov AK Sander T Ramirez A Poser B Maljevic S Hebeisen S Kubisch C Rebstock J Mutations in CLCN2 encoding a voltage-gated chloride channel are associated with idiopathic generalized epilepsies. Nat Genet 2003 33 527 532 10.1038/ng1121 12612585

[B29] Chioza B Wilkie H Nashef L Blower J McCormick D Sham P Asherson P Makoff AJ Association between the alpha(1a) calcium channel gene CACNA1A and idiopathic generalized epilepsy. Neurology 2001 56 1245 1246 11342703 10.1212/wnl.56.9.1245

[B30] Chen Y Lu J Pan H Zhang Y Wu H Xu K Liu X Jiang Y Bao X Yao Z Association between genetic variation of CACNA1H and childhood absence epilepsy. Ann Neurol 2003 54 239 243 10.1002/ana.10607 12891677

[B31] Robbins TW Murphy ER Behavioural pharmacology: 40+ years of progress, with a focus on glutamate receptors and cognition. Trends Pharmacol Sci 2006 27 141 148 10.1016/j.tips.2006.01.009 16490260 PMC1867319

[B32] Zamanillo D Sprengel R Hvalby O Jensen V Burnashev N Rozov A Kaiser KM Koster HJ Borchardt T Worley P Importance of AMPA receptors for hippocampal synaptic plasticity but not for spatial learning. Science 1999 284 1805 1811 10.1126/science.284.5421.1805 10364547

[B33] Schmitt WB Sprengel R Mack V Draft RW Seeburg PH Deacon RM Rawlins JN Bannerman DM Restoration of spatial working memory by genetic rescue of GluR-A-deficient mice. Nat Neurosci 2005 8 270 272 10.1038/nn1412 15723058

[B34] Schmitt WB Deacon RM Seeburg PH Rawlins JN Bannerman DM A within-subjects, within-task demonstration of intact spatial reference memory and impaired spatial working memory in glutamate receptor-A-deficient mice. J Neurosci 2003 23 3953 3959 12736365 10.1523/JNEUROSCI.23-09-03953.2003 PMC6742186

[B35] Campion D Flaman JM Brice A Hannequin D Dubois B Martin C Moreau V Charbonnier F Didierjean O Tardieu S Mutations of the presenilin I gene in families with early-onset Alzheimer's disease. Hum Mol Genet 1995 4 2373 2377 10.1093/hmg/4.12.2373 8634712

[B36] Stutzmann GE Caccamo A LaFerla FM Parker I Dysregulated IP3 signaling in cortical neurons of knock-in mice expressing an Alzheimer's-linked mutation in presenilin1 results in exaggerated Ca2+ signals and altered membrane excitability. J Neurosci 2004 24 508 513 10.1523/JNEUROSCI.4386-03.2004 14724250 PMC6729995

[B37] Sommer B Keinanen K Verdoorn TA Wisden W Burnashev N Herb A Kohler M Takagi T Sakmann B Seeburg PH Flip and flop: a cell-specific functional switch in glutamate-operated channels of the CNS. Science 1990 249 1580 1585 10.1126/science.1699275 1699275

[B38] Seifert G Zhou M Dietrich D Schumacher TB Dybek A Weiser T Wienrich M Wilhelm D Steinhauser C Developmental regulation of AMPA-receptor properties in CA1 pyramidal neurons of rat hippocampus. Neuropharmacology 2000 39 931 942 10.1016/S0028-3908(99)00212-9 10727703

[B39] Seifert G Huttmann K Schramm J Steinhauser C Enhanced relative expression of glutamate receptor 1 flip AMPA receptor subunits in hippocampal astrocytes of epilepsy patients with Ammon's horn sclerosis. J Neurosci 2004 24 1996 2003 10.1523/JNEUROSCI.3904-03.2004 14985442 PMC6730392

[B40] Pollard H Heron A Moreau J Ben-Ari Y Khrestchatisky M Alterations of the GluR-B AMPA receptor subunit flip/flop expression in kainate-induced epilepsy and ischemia. Neuroscience 1993 57 545 554 10.1016/0306-4522(93)90004-Y 8309523

[B41] Helton TD Horne WA Alternative splicing of the beta 4 subunit has alpha1 subunit subtype-specific effects on Ca2+ channel gating. J Neurosci 2002 22 1573 1582 11880487 10.1523/JNEUROSCI.22-05-01573.2002 PMC6758875

[B42] Liu H De Waard M Scott VE Gurnett CA Lennon VA Campbell KP Identification of three subunits of the high affinity omega-conotoxin MVIIC-sensitive Ca2+ channel. J Biol Chem 1996 271 13804 13810 10.1074/jbc.271.23.13804 8662888

[B43] Heinzen EL Yoon W Tate SK Sen A Wood NW Sisodiya SM Goldstein DB Nova2 interacts with a cis-acting polymorphism to influence the proportions of pharmacologically relevant splice variants of SCN1A. Am J Hum Genet 2007 in press 10.1086/516650 PMC1852745 17436242

[B44] Tate SK Singh R Hung C Tai JJ Depondt C Cavalleri GL Sisodiya SM Goldstein DB Horng-Huei L A common polymorphism in the SCN1A gene associates with phenytoin serum levels at maintenance dose. Pharmacogenet Genomics 2006 16 721 726 17001291 10.1097/01.fpc.0000230114.41828.73

[B45] Hulette C Welsh-Bohmer K Crain B Szymanski M Sinclaire N Roses A Rapid brain autopsy: The Brayn Alzheimer's Disease Research Center experience. Arch Pathol Lab Med 1997 121 615 618 9199629

[B46] Applied Biosystems. Guide to performing relative quantitation of gene expression using real-time quantitative PCR. http://docs.appliedbiosystems.com/pebiodocs/04371095.pdf

