# Supplementary material for: Alternative ion channel splicing in mesial temporal lobe epilepsy and Alzheimer's disease
Source: Genome Biol. 2007 Mar 7;8(3):R32. doi: 10.1186/gb-2007-8-3-r32 (PMC1868939; doi:10.1186/gb-2007-8-3-r32)
Supplement: Additional data file 1 — Taqman® assays and custom-designed primer/probe sequences used in rtPCR studies to confirm the presence of the splice variants. [file gb-2007-8-3-r32-S1.doc]

***Additional Data File 1. Taqman® real time PCR assays (Applied Biosystems)***

| ***Gene*** | **Assay sequence for reference transcript** | **Assay sequence for variant transcript** |
| --- | --- | --- |
| ***CACNA1B*** | **NM_000718**  ABI assay: Hs00609489_g1 | **M94173**  Forward: CCCAACGTCGCCAACAG  Reverse: GGCGTTGTGTTCGGAAAGC  Probe: FAM-CCCCACCCACAGGCC-NFQ |
| ***CACNA1G*** | **NM_018896**  Forward: GCAAGATCTCCAAGCACATGAC  Reverse: TGCCCCAGTTGGGTTCTG  Probe: CCAGCCCCTTGCCC | **NM_198376**  Forward: GACTGACTCCTTGGACGTTCA  Reverse: CACGCTGTAGCACTTCTTCAG  Probe: CTCTGCCAGCAGGTCT |
| ***CACNB1*** | **NM_000723**  ABI assay: Hs00609505_m1 | **NM_199247**  Forward: CCTCCAATTCTTCCTCCTTTTAGCT  Reverse: TGGACGGCTGGTGTAGGA  Probe: FAM-CCAGCTCTGATCCCCG-NFQ |
| ***CACNB4*** | **NM_000726**  ABI assay: Hs00947802_m1 | **AY054985**  Forward: TGTATGACAATTTGTACCTGCATGGA  Reverse: AGACGGCCTGCTTGTGTAG  Probe: FAM-CTGGTTCAGCGGATTC-NFQ |
| ***CLCN7*** | **NM_001287**  Forward: CGGCAAATACGCCCTGATG  Reverse: CGGTCAGGCTCAGTGTCA  Probe: FAM-TCCGCACAATCCCGCC-NFQ | **AK096963**  Forward: GCTCTTGCCCGATGGGT  Reverse: CCTAGCCCCGCAAGGA  Probe: FAM-CAGCCATGGCCCCC-NFQ |
| ***FXYD1*** | **NM_021902**  Forward: CGCAGGCGGTAGAAACAC  Reverse: GGAGCGTGGGAGATGTCA  Probe: FAM-CTGGAGCGATGGAATC-NFQ | **BX447949**  Forward: GCCAAGTGCCAGAGTTGAAG  Reverse: GTGAGGAGTCTTTGAGGGTGAAAA  Probe: FAM-ACGTCAGCCCTCGCCGC-NFQ |
| ***FXYD6*** | **NM_022003**  ABI assay: Hs01121137_m1 | **BP372334**  Forward: CCAGGGAGGCAGCAAGA  Reverse: GCGTCTGGAACTGTCACTAATTCTG  Probe: FAM-CATGCACCTTCCACTCTT-NFQ |
| ***GABRA6*** | **NM_000811**  Forward: GATTGGACAAACAGTATCTAGTGAGACA  Reverse: CTTTGCAAGTGGAAGTAAACTGTCA  Probe: FAM-TATTCACCTGTGTTAGATTTAA-NFQ | **AK090735**  Forward: GGTGCATATAAAGATTTGTGTAATCTTGGAATAA  Reverse: GATAGAGCGAAGAGACACATCAACT  Probe: FAM-TTGTTTTCAGTAGGGTTCTTTATATC-NFQ |
| ***GRIA1*** | **NM_000827**  Forward: ACGACAAGGGCGAGTGC  Reverse: ACGCCTGCCACATTGCT  Probe: FAM-TTGTCCTTGGAATCACC-NFQ | **A46050**  Forward: CCTAGCGGTTTTGAAACTCAGTGA  Reverse: CCTTTATCGTACCACCATTTGCTTT  Probe: FAM-AAGGCGTCTTAGACAAGC-NFQ |
| ***GRIA3*** | **NM_007325**  Forward: GCCTGTAAACCTTGCAGTATTGAAA  Reverse: CCCCTTATCGTACCACCATTTGTTT  Probe: FAM-TCAGCTTGTCTAAGATGCC-NFQ | **NM_000828**  Forward: AACCCCTAAAGGCTCAGCATT  Reverse: CCTTTGTCGTACCACCATTTGTTTT  Probe: FAM-CCTCTTGGACAAATTG-NFQ |
| ***HTR3A*** | **NM000869**  ABI assay: Hs00356082_m1 | **BG341613**  Forward: CCAGATCTCTGCTTTCCTGTCA  Reverse: CGGGCCTGGTGGTGTTT  Probe: FAM- CAAGCCAGGAGGAGCC-NFQ |
| ***KCNAB1*** | **NM_172160**  ABI assay: Hs00967804_m1 | **NM_172159**  Forward: TCCAGCTGCTGTGACAACTTC  Reverse: GCTGCGATGCAGGAAAGAG  Probe: FAM-CTGTTTACTTCTCTGGGTCCC-NFQ |
| ***KCNIP2*** | **NM_014591**  Forward: CAAGCCCTGCCCTCAGT  Reverse: GTCACCGAGAAAGCGGAAGA  Probe: FAM-CCCGGCCAATTTCACT-NFQ | **NM_173197**  Forward: CCTGGACGGCTCCTACGA  Reverse: CGGTGGACAATTCAAATTCATCGT  Probe: FAM-ACGCTGTCCGTGAGCTG-NFQ |
| ***KCNK1*** | **NM_002245**  ABI assay: Hs01116799_m1 | **AV733795**  Forward: CCCTTCACCCTCCTGTTCCT  Reverse: GCCGGGATGAAGAAGAAGCA  Probe: FAM-TCCAGCGCATCACTG-NFQ |
| ***KCNN1*** | **NM_002248**  Forward: GTAAGTTCCTCCAAGCCATCCAT  Reverse: CCCTTGCTCGATCTTCACACT  Probe: FAM-CAGGCTCAGAAGCTC-NFQ | **BM718136**  Forward: AAGCCCGGGTTCGGAAA  Reverse: CCCTTGCTCGATCTTCACACT  Probe: FAM-CACCAGCGGCTCAGAAG-NFQ |
| ***KCNN2*** | **NM_021614**  Forward: CCACCGCTGATGTGGATATTATTTTATCTATA  Reverse: GGCATCAGTGAAAAGTTTGCTATGT  Probe: FAM-CATGACTCTGGCAATCAG-NFQ | **BG769522**  Forward: TCGGTCTGATCATCGTGTACCA  Reverse: CGCTCCAAGGAAGTTGCTAGTAA  Probe: FAM-ATCATGGTACCTGTATTTCC-NFQ |
| ***KCNQ2*** | **NM_004518**  ABI assay: Hs01548344_m1 | **AY358189**  Forward: TCTGGGTCCAGCCTACCTTAA  Reverse: CCGTCAATAGTGGAGTGAGATTTGG  Probe: FAM-TTCCACTATAAAAACTACAAACAGC-NFQ |
| ***MCOLN1*** | **NM_020533**  ABI assay: Hs01100661_g1 | **CA489568**  Forward: CCCAGGCCCACATCCA  Reverse: GGGAGCAGGTGAGGATGAC  Probe: FAM-CCGGAAGCTGTTGTCTCA-NFQ |
| ***SCN1A*** | **NM_006920**  Forward: AACTGGCTCGATTTCACTGTCATTA  Reverse: ATTGCCCAGGTCCACAAACT  Probe: FAM-CTGTGACGTACGCAAATG-NFQ | **AX164172**  Forward: TGGCTCGATTTCACTGTCATTACAT  Reverse: TCTGAAAGTGCGAAGAGCTGAAAA  Probe: FAM-CCTAGGTTTACAAATTCTG-NFQ |
|  |  |  |
