# Supplementary material for: Alternative ion channel splicing in mesial temporal lobe epilepsy and Alzheimer's disease
Source: Genome Biol. 2007 Mar 7;8(3):R32. doi: 10.1186/gb-2007-8-3-r32 (PMC1868939; doi:10.1186/gb-2007-8-3-r32)
Supplement: Additional data file 2 — Additional methods, discussion and figures regarding the assessment of cellular composition of the brain tissue samples used in these studies. [file gb-2007-8-3-r32-S2.doc]

**Additional Data File 2**

*Supplemental Results/Discussion:*

In conducting these experiments, we anticipated seeing a decrease in neuronal cellular composition (decreased NeuN staining) and an increase in the glial cell population (GFAP staining) in the brain tissue samples collected from AD and mTLE samples, as these changes are consistent with neurodegeneration. However, neuronal cell quantities were found to be increased in both disease states when compared to control (Additional data figure 1). This NeuN protein expression correlated with the time of brain tissue collection. For mTLE patients, brain tissue was removed and immediately flash frozen, while in the control and AD subjects the tissue was collected postmortem with times of collection ranging from 0.5-30 hrs. Specifically the AD samples were collected on average 1.5 hrs earlier than control samples (8 v 9.5 hrs in control and AD, respectively), while the mTLE samples were freshly resected. This temporal difference is in good relationship between the observed changes in NeuN protein expression, indicating that the NeuN protein may be degrading in the time elapsed between death and brain tissue collection. Therefore, the utility of this neuronal cell marker in this work is questionable. However, GFAP exhibited an increase in protein expression of approximately the same magnitude in both AD and epileptic samples when compared to controls, a finding consistent with neurodegeneration characteristic of these disease states. Importantly, the increase in glial cells was not correlated with the extent of splicing proportions of any of the identified splicing changes reported here (Additional data figure 2). Furthermore, due to concerns of differential transcript degradation of splice variants, a linear regression analysis was performed on a percentage of splice variants versus time of brain tissue collection for the identified splicing changes. The time of brain tissue collection did not correlate with ratios of splice variants that were found to be changed in the disease states.

*Supplemental Materials and Methods:*

As both AD and mTLE can involve neuronal loss (and glial cell proliferation), immunoblot analyses were conducted to ensure that any observed splicing changes were not due to an alteration in cellular populations. Tissue samples were immunoblotted for NeuN (a neuronal cell marker), GFAP (a glial cell marker), and β-actin (loading control). Initially, the protein content in the samples was analyzed using a western blot procedure (separation followed by immunoblotting), however we found that in all cases the resultant blot produced only one band, or set of bands, representing the protein(s) of interest. Furthermore, a direct comparison of immunoblots with and without protein separation yielded equivalent results. Therefore, straightforward immunoblotting without protein separation was performed on each brain tissue sample.

Samples of frozen ground tissue were lysed in 2% SDS in PBS containing protein inhibitors (Sigma, P8340). A single concentrated sample from each of mTLE, control, and AD temporal cortical tissue was used to create a standard curve (2-40 µg protein/2 µL). 10 – 20 micrograms of mTLE, control, and AD temporal cortical tissue homogenate samples were loaded onto a nitrocellulose membrane. The membrane was blocked for 1 hr in 5% milk in TBST solution and then incubated overnight at 4^○^C with primary antibodies. Primary antibodies used were the following: rabbit anti-β-actin antibody (Abcam, AB8227) 1:2,000 dilution, mouse anti-GFAP (Chemicon, CBL411) 1:4,000 dilution and mouse anti-NeuN (Chemicon, MAB377) 1:1,000 dilution. Blots were then washed, incubated with species-specific peroxidase-conjugated secondary antibodies and developed using ECL. Blots were then stripped and sequentially reprobed with the remaining two primary antibodies. Images from the blots were digitialized using HP ScanJet 4890. Densitometric analysis was performed using ImageJ software. GFAP and NeuN protein amounts were normalized to β-actin. NeuN and GFAP protein amounts were compared between control temporal cortex, mTLE neocortex, and AD temporal cortex for all available samples. Linear regression of splice variant proportions for all identified events (in the splice array and in the rtPCR follow-up samples) was performed individually comparing the extent of alternative splicing for a given event versus NeuN and GFAP protein levels.

*

*

*

*

***Additional data figure 1.*** ***Quantitation of cellular composition in brain tissue samples.***  Comparison of NeuN and GFAP protein expression (normalized to β-actin expression) in control (Control TC), mTLE (mTLE NC), and AD (AD TC) brain tissue.

Data are presented mean ± SEM.

*p<0.001 compared to control temporal cortex, one-way ANOVA, Tukey posthoc statistical analyses*

***Additional data figure 2.***  ***Representative graph depicting the lack of correlation between protein expression and SVRs.*** Correlation between *MCOLN* splice variant ratio (SVR) and NeuN (A.) and GFAP (B.) protein expression in temporal cortical tissue collected for control (○), mTLE (●), and AD (▼) subjects. Solid line indicates the line fitted to the data using linear regression. No statistical correlation was observed.
